# Expression and Function of the Cholinergic System in Immune Cells

**DOI:** 10.3389/fimmu.2017.01085

**Published:** 2017-09-06

**Authors:** Takeshi Fujii, Masato Mashimo, Yasuhiro Moriwaki, Hidemi Misawa, Shiro Ono, Kazuhide Horiguchi, Koichiro Kawashima

**Affiliations:** ^1^Faculty of Pharmaceutical Sciences, Department of Pharmacology, Doshisha Women’s College of Liberal Arts, Kyoto, Japan; ^2^Faculty of Pharmacy, Department of Pharmacology, Keio University, Tokyo, Japan; ^3^Laboratory of Immunology, Faculty of Pharmacy, Osaka Ohtani University, Osaka, Japan; ^4^Department of Anatomy, Division of Medicine, University of Fukui Faculty of Medical Sciences, Fukui, Japan; ^5^Department of Molecular Pharmacology, Kitasato University School of Pharmaceutical Sciences, Tokyo, Japan

**Keywords:** dendritic cell, lymphocyte, macrophage, mAChR, nAChR, SLURP-1

## Abstract

T and B cells express most cholinergic system components—e.g., acetylcholine (ACh), choline acetyltransferase (ChAT), acetylcholinesterase, and both muscarinic and nicotinic ACh receptors (mAChRs and nAChRs, respectively). Using ChAT^BAC^-eGFP transgenic mice, ChAT expression has been confirmed in T and B cells, dendritic cells, and macrophages. Moreover, T cell activation *via* T-cell receptor/CD3-mediated pathways upregulates ChAT mRNA expression and ACh synthesis, suggesting that this lymphocytic cholinergic system contributes to the regulation of immune function. Immune cells express all five mAChRs (M_1_–M_5_). Combined M_1_/M_5_ mAChR-deficient (M_1_/M_5-_KO) mice produce less antigen-specific antibody than wild-type (WT) mice. Furthermore, spleen cells in M_1_/M_5_-KO mice produce less tumor necrosis factor (TNF)-α and interleukin (IL)-6, suggesting M_1_/M_5_ mAChRs are involved in regulating pro-inflammatory cytokine and antibody production. Immune cells also frequently express the α2, α5, α6, α7, α9, and α10 nAChR subunits. α7 nAChR-deficient (α7-KO) mice produce more antigen-specific antibody than WT mice, and spleen cells from α7-KO mice produce more TNF-α and IL-6 than WT cells. This suggests that α7 nAChRs are involved in regulating cytokine production and thus modulate antibody production. Evidence also indicates that nicotine modulates immune responses by altering cytokine production and that α7 nAChR signaling contributes to immunomodulation through modification of T cell differentiation. Together, these findings suggest the involvement of both mAChRs and nAChRs in the regulation of immune function. The observation that vagus nerve stimulation protects mice from lethal endotoxin shock led to the notion of a cholinergic anti-inflammatory reflex pathway, and the spleen is an essential component of this anti-inflammatory reflex. Because the spleen lacks direct vagus innervation, it has been postulated that ACh synthesized by a subset of CD4^+^ T cells relays vagal nerve signals to α7 nAChRs on splenic macrophages, which downregulates TNF-α synthesis and release, thereby modulating inflammatory responses. However, because the spleen is innervated solely by the noradrenergic splenic nerve, confirmation of an anti-inflammatory reflex pathway involving the spleen requires several more hypotheses to be addressed. We will review and discuss these issues in the context of the cholinergic system in immune cells.

## Introduction

Acetylcholine (ACh) is one of the old neurotransmitters identified in the central and peripheral nervous systems. First synthesized in 1867 by von Baeyer, who acetylated choline using acetylchloride, ACh was left on the chemical list for several decades without exploration of its biological activity [see a review by Burgen (1)]. In 1914, however, Ewins (2) identified ACh as the active principle in ergot that exerts an inhibitory effect on the heart but a stimulatory effect on intestinal muscle. This was the first discovery of ACh in a life form. Those findings prompted Dale (3) to extensively investigate the biological activities of choline derivatives, including ACh. A little later, Loewi (4) demonstrated that the effects of autonomic nerve impulses were transmitted through peripheral release of a specific chemical stimulant in isolated frog heart preparations; this was later proved pharmacologically to be ACh (5). Dale and Dudley (6) then successfully isolated ACh from the spleens of an ox and a horse, making them the first to isolate ACh from an animal organ. On the basis of these findings, and in conjunction with the resemblance between the effects of sympathetic nerves and those of adrenaline (7), Dale (8) suggested the term “cholinergic” to describe nerves that transmit their action through release of ACh, and “adrenergic” for those who employ a substance resembling adrenaline. In 1936, The Nobel Prize in Physiology or Medicine was awarded jointly to Sir Henry H. Dale and Otto Loewi “for their discoveries relating to chemical transmission of nerve impulses.” Since then, ACh has been widely recognized as a neurotransmitter.

The expression of muscarinic and nicotinic ACh receptors (mAChRs and nAChRs, respectively) in lymphocytes and thymocytes has been known since early 1970s, based on the various functional and biochemical changes elicited by ACh and agonists such as carbachol, oxotremorine (Oxo), and nicotine in these cells [see a review by Kawashima and Fujii (9)]. Moreover, expression of mAChRs and nAChRs in lymphocytes and thymocytes was confirmed in binding studies using radiolabeled mAChR and nAChR ligands such as [^3^H]quinuclinidyl benzilate, [^3^H]nicotine, and [^125^I]α-bungarotoxin (α-BTX) [see reviews in Ref. (9–12)]. At the time of its discovery, the origin of the splenic ACh isolated by Dale and Dudley (6) was left unexplored, and the findings summarized above were interpreted to show control of immune cells by the parasympathetic nervous system *via* ACh. Although the anatomy of immune system innervation has not yet been fully described, it is now generally agreed that the spleen receives innervation by sympathetic neurons but not by parasympathetic cholinergic neuron (10, 13–16). And the enigma of the origin of ACh that should act on the mAChRs and nAChRs on immune cells was ultimately solved based on the discovery of ACh in the blood and its localization to lymphocytes using a sensitive and specific radioimmunoassay for ACh (17, 18); also see reviews (9, 11, 12). Thereafter, data from a variety of investigations provided evidence that immune cells possess all the required components to constitute an independent cholinergic system, including choline acetyltransferase (ChAT, EC 2.3.2.6) and acetylcholinesterase (AChE, EC 3.1.1.7) as well as mAChRs and nAChRs [see reviews in Ref. (9, 11, 12)]. Furthermore, as reviewed by Fujii et al. (16), recent findings on the cholinergic system in immune cells suggest that ACh synthesized by immune cells plays a key role in the regulation of immune function by triggering signals that initiate and terminate cytokine production in immune cells.

In this review, we will discuss (1) the cholinergic components expressed in T and B cells, macrophages, and dendritic cells (DCs); (2) the functions of AChRs in the regulation of immune cell activity; and (3) the functions of the immune cell cholinergic system within an anti-inflammatory reflex.

## Cholinergic Components Expressed in Immune Cells

We will first discuss the following major cholinergic system components: (1) ACh and ChAT, an ACh-synthesizing enzyme; (2) the ACh-degrading enzymes AChE and butyrylcholinesterase (BuChE, EC 3.1.1.8); (3) mAChRs and nAChRs; and (4) secreted lymphocyte antigen-6/urokinase-type plasminogen activator (SLURP)-1 and -2, two endogenous positive allosteric ligands for α7 and α3 nAChRs.

### ACh and ChAT

In immune cells and in the central and peripheral nervous systems, ACh is synthesized from choline and acetyl coenzyme A (acetyl-CoA) by ChAT.

#### ACh in Immune Cells

After discovery of ACh in the peripheral blood and plasma of humans and animals, the presence of ACh in immune cells was first demonstrated in the human peripheral blood mononuclear leukocyte (MNL) fraction, which consists mainly of lymphocytes and a small monocyte fraction (18–23); also see a review (9). Later, the presence of ACh in immune cells was confirmed by detection of ACh in various human leukemic cell lines (24, 25) and rat lymphocytes, including T and B cells (26). These findings provided an explanation for the seemingly enigmatic observation of Dale and Dudley (6) that ACh was present in the spleen, though that organ is not cholinergically innervated.

In general, human leukemic T cell lines had higher ACh contents than B cell lines, prelymphoma cell lines, or a monocytic cell line (Table 1) (9, 25). Among rat lymphocytes, the ACh content in T cells was significantly higher than in B cells, and the ACh content in CD4^+^ T cells was significantly higher than in CD8^+^ T cells (26). The higher ACh content observed in rat T cells than B cells reflects the higher ChAT activity in T cells (27). Little information is available on the intracellular ACh contents in macrophages and DCs.

**Table 1:** ACh content, ChAT and CarAT activities, and ChAT mRNA expression in human leukemic cell lines.

Cell line	Cell type	ACh content pmol/10^6^ cells	ChAT activity pmol/mg protein/min	CarAT activity pmol/mg protein/min	ChAT mRNA expression
CEM	T	12.6 ± 0.6	2.9 ± 0.2	22.8 ± 4.6	Positive
HSB-2	T	36.2 ± 3.5	1.4 ± 0.1	58.3 ± 15.3	Positive
Jurkat	T	8.2 ± 0.4	4.3 ± 0.8	17.2 ± 1.9	Positive
MOLT-3	T	251.5 ± 34.9	22.4 ± 3.0	53.3 ± 5.6	Positive
MOLT-4	T	38.8 ± 5.9	8.0 ± 1.0	NT	Positive
BALL-1	B	ND	0.4 ± 02	NT	Negative
Daudi	B	1.2 ± 0.1	0.1 ± 0.02	125.6 ± 44.2	Negative
NALM-6	B	0.04 ± 0.01	0.1 + 0.02	NT	Negative
REH	Pre lymphoma	0.8 ± 0.01	0.2 ± 0.03	NT	Negative
U937	Monocytic	0.02 ± 0.01	0.2 ± 0.03	NT	Negative

*Values are mean ± SEM*.

*ND, not detectable; NT, not tested*.

*ChAT activity was calculated from the difference in the ACh-synthesizing activities in the presence and absence of 100 µM bromoacetylcholine. CarAT activity was calculated from the difference in the Ach-synthesizing activities in the presence and absence of 100 μM bromoacetylcamitine. Data arranged from the studies by Fujii et al. (25) and Kawashima and Fujii (9)*.

It is important to note that, with the molecular weight of 146 Da, ACh is small, water soluble, and both physicochemically and enzymatically fragile. Furthermore, the chemical nature of ACh is quite different from that of catecholamines and serotonin, which are able to be fixed to a tissue using paraformaldehyde. At present, no technique is available to fix ACh to the tissue. It is therefore currently impossible to detect ACh in tissues or cells using immunohistochemical or immunocytochemical techniques. Although ACh may bind to an antibody, because it is not anchored to its site, it will be washed away with the antibody. Nonetheless, Takahashi et al. (28) reported a successful attempt to localize ACh in the mouse gut sections using a tandem imaging mass spectrometry.

#### ChAT in Immune Cells

Kajiyama et al. (29) observed that a major portion of rabbit blood ACh is present in the buffy coat along with ACh-synthesizing activity. On the basis of that observation, Rinner and Schauenstein (27) confirmed the expression of ACh-synthesizing activity in rat T and B cells in the thymus, spleen, and blood, suggesting the presence of ChAT in lymphocytes.

ChAT is primarily responsible for ACh synthesis within the nervous systems of mammalian species (30). However, in peripheral tissues and non-neuronal cells, the mitochondrial enzyme carnitine acetyltransferase (CarAT, EC 2.3.1.7) also contributes to ACh synthesis along with ChAT (30, 31). ACh-synthesizing activity determined in peripheral samples using the so-called Fonnum method (32) with [^3^H]acetyl coenzyme A and choline reflects the total activities of ChAT and CarAT. It is therefore recommended that one determines ChAT and CarAT activities in the presence of respective specific inhibitors, bromoacetylcholine and bromoacetylcarnitine. ChAT activity is proportional to the ACh content in cells of the T cell lines expressing ChAT mRNA (Table 1) (10, 25). However, Daudi B cells, which do not express ChAT mRNA, contained little ACh despite of a high CarAT activity. This suggests that ChAT is responsible for ACh synthesis in immune cells (10).

#### Expression of ChAT Enzyme Protein and mRNA

**Figure 1:**
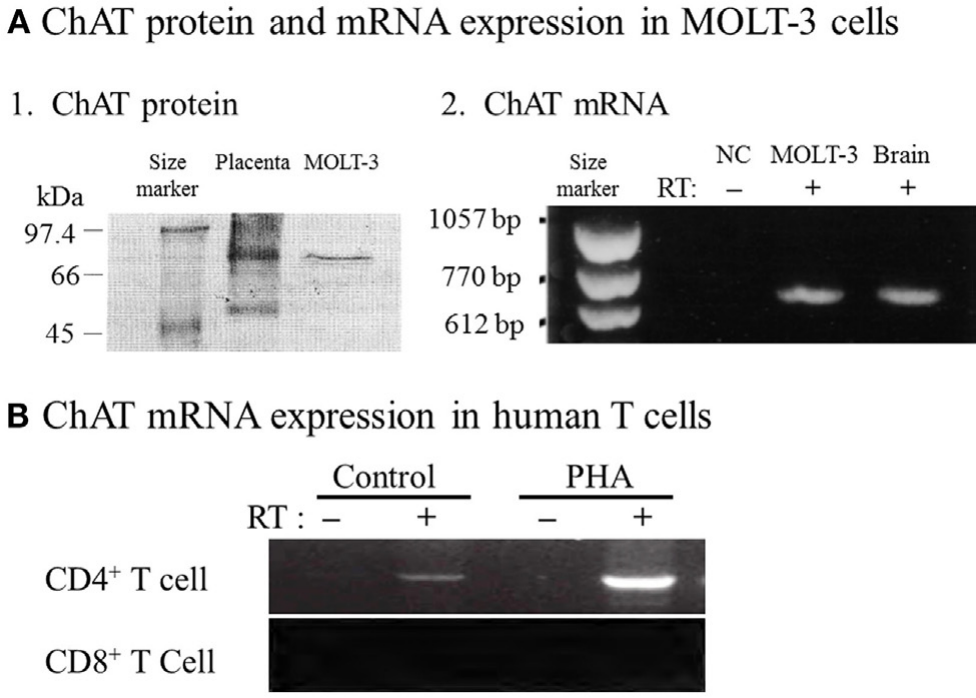
Expression of choline acetyltransferase (ChAT) mRNA and protein in human immune cells. **(A1)** Western blot analysis of ChAT protein expression in MOLT-3 human leukemic T cells. **(A2)** Expression of ChAT mRNA detected using reverse transcription-polymerase chain reaction (RT-PCR). NC, negative control of MOLT-3 without RT. Arranged from study by Fujii et al. (33). **(B)** Expression of ChAT mRNA in human CD4^+^ T cells and its potentiation by immunological activation with phytohemagglutinin (PHA). Note that CD8^+^ T cells do not express ChAT mRNA, even after immunological activation. RT, reverse transcriptase. Arranged from study by Fujii et al. (34).

Fujii et al. (33) provided definitive evidence for the synthesis of ACh by ChAT in T cells by demonstrating expression of ChAT mRNA and the enzyme protein in MOLT-3 human leukemic T cells, using reverse transcription-polymerase chain reaction (RT-PCR) and western blot analysis (Figure 1A). Later, constitutive ChAT mRNA expression was detected in other human leukemic T cell lines (25), human blood CD4^+^ T cells (Figure 1B) (34), rat T and B cells (24, 26), and rat MNLs isolated from the renal vasculature (35). These findings support the idea that ACh production catalyzed by ChAT is occurring in lymphocytes, including T and B cells.

Reverse transcription-polymerase chain reaction revealed ChAT mRNA expression in C57BL/6J mouse spleen-derived MNLs activated with concanavaline A (ConA) and bone marrow-derived DCs activated with lipopolysaccharide (LPS) (36). However, there was no detectable expression of ChAT mRNA in these cells under resting conditions, which suggests that immunological activation is required for ChAT transcription in these cells. While no ChAT mRNA was detected in peritoneal macrophages under either resting or LPS-activated conditions, even after an amplification protocol entailing 40 cycles (36), Koarai et al. (37) detected expression of ChAT mRNA in human lung and alveolar macrophages and monocytes using an RT-PCR protocol entailed 45 cycles, which suggests marginal ChAT mRNA expression in these cells. ChAT mRNA and protein were also detected in human mature and immature DCs using RT-PCR and immunocytochemistry (38). These findings confirm the expression of ChAT mRNA in T and B cells, DCs, and macrophages.

#### Expression of a Fluorescent ChAT-Reporter Protein

The recent development of ChAT^BAC^-eGFP transgenic mice (39) and ChAT-Cre-tdTomato mice (40) provide the opportunity to detect ChAT-expressing cells using fluorescent reporter proteins. Tallini et al. (39) observed eGFP expression in a subset of lymphocytes in Peyer’s patches, leading to the detection of ChAT-GFP in splenic CD4^+^ T cells (15) and CD8^+^ T cells and B cells (41). In addition, Gautron et al. (40) observed expression of the reporter protein in T cells within Peyer’s patches in mice expressing tdTomato fluorescent protein in ChAT-expressing cells. These results confirm the earlier findings that peripheral blood CD4^+^ and CD8^+^ T cells and B cells express ChAT mRNA and contain ACh (26, 36).

ChAT expression in splenic DCs was confirmed using ChAT^BAC^-eGFP mice (41). That finding along with the ChAT gene expression in DCs described above (36) suggests DCs are able to synthesize ACh using ChAT. So far, however, no additional data on ACh synthesis and release in DCs has been reported, and the physiological significance of ACh in DCs is yet to be determined.

ChAT expression was also observed in splenic macrophages using ChAT^BAC^-eGFP transgenic mice (41). These findings are in line with those from Koarai et al. (37), who reported the expression of ChAT mRNA in human lung and alveolar macrophages and monocytes. However, Gautron et al. (40) did not find reporter expression in macrophages from gut-associated lymphoid tissue or the spleen of ChAT-Cre-tdTomato mice. As mentioned, we did not detect ChAT mRNA expression in either resting or activated mouse peritoneal macrophages (36). These findings suggest ChAT expression in macrophages may vary depending upon strain, species, tissue, cell processing procedure, and/or immunological status, or that the levels of ChAT expression are marginal in macrophages. Information on the physiological significance of ACh synthesis in macrophages is not available at present.

#### Regulatory Mechanisms Affecting ChAT Expression and ACh Synthesis in Immune Cells

##### Lymphocytes

###### *T* *Cells*

Fujii et al. (24) first suggested a role for ACh synthesized by T cells in the regulation of immune system function by showing that phytohemagglutinin (PHA), a T cell activator, increased both intracellular ACh content and its release into the culture medium of HSB-2 and MOLT-3 human leukemic T cells used as models of T cells. In human MNLs, PHA activates protein kinase C (PKC) and mitogen-activated protein kinase (MAPK) pathways *via* the T-cell receptor (TCR)/CD3 complex, resulting in specific upregulation of ChAT mRNA expression, ChAT activity, and ACh synthesis (42). However, PHA does not upregulate CarAT activity, indicating that only ChAT is linked to T cell activity (42). Upregulation of ChAT mRNA expression by T cell activation with PHA or Con A was also confirmed in rat T cells (26) and mouse spleen cells (36). These findings support the notion that immunological activation of T cells upregulates ACh synthesis, leading to modulation of immune function.

Antithymocyte globulin (ATG)-Fresenius, an immunosuppressant that binds to cell surface molecules, including CD2, CD3, CD4/CD28, CD5, CD7, CD11a (lymphocyte function-associated antigen (LFA)-1), and intercellular adhesion molecule (ICAM)-1, increases ACh release but decreases intracellular ACh content in CCRF-CEM (CEM) human leukemic T cells in the short term (6 h), and increases both ACh release and intracellular ACh content in the long term (48 h) (43). Both anti-CD11a monoclonal antibody (mAb) and ATG-F upregulates ChAT mRNA expression after 48 h of culture, suggesting activation of T cell adhesion molecules facilitates ACh synthesis (43). By contrast, acting in a manner independent of hydroxymethylglutaryl-CoA reductase inhibition, statins, including simvastatin, are able to inhibit LFA-1 (CD11a/CD18)-mediated adhesion and co-stimulation of lymphocytes, leading to immune modulation (44). Simvastatin thus abolishes anti-CD11a mAb-induced increases in ChAT mRNA expression, ACh synthesis and release in MOLT-3 cells (45). These results confirm that cell adhesion molecules such as LFA-1 contribute to the regulation of lymphocytic cholinergic activity (45, 46).

Calcium ionophores such as A23187 and ionomycin upregulate expression of ChAT mRNA and its activity, and they increase the ACh content of MOLT-3 human leukemic T cells and their culture media (46, 47). By contrast, FK-506 (tacrolimus), a calcineurin inhibitor, suppresses PHA-induced upregulation of ChAT mRNA expression and ACh synthesis, which suggests that Ca^2+^ contributes to the regulation of T cell cholinergic activity through calcineurin-mediated pathways (46).

Phorbol 12-myristate 13-acetate (PMA), a non-specific PKC activator, and dibutyryl cAMP, a protein kinase A (PKA) activator, increased ChAT activity and ACh synthesis by upregulating ChAT gene expression in MOLT-3 human leukemic T cells (46). These data provide compelling evidence that T-cell activation *via* PKC–MAPK and/or adenylate cyclase-cAMP pathways during immune responses upregulates the synthesis and release of ACh, leading to the modulation of the T cell cholinergic activity.

###### *B* *Cells*

*Staphylococcus aureus* Cowan I (SAC) binds to B cells and triggers a signal transduction cascade involving tyrosine kinase-mediated activation of phospholipase C (PLC) and leads to activation of PKC–MAPK pathways (48). Incubation of human circulating MNLs consisting of mainly T and B cells and a small number of monocytes with SAC for 48 h induces a significant increase of the intracellular ACh content and upregulation of ChAT mRNA expression (47). These findings suggest that stimulation of B cells also facilitates cholinergic activity *via* upregulation of ChAT mRNA expression.

Murine B cells, but not human B cells, express toll-like receptor 4 (TLR-4) (49). LPS activates murine B cells, monocytes, DCs, and macrophages by binding to a CD14/TLR-4/MD2 complex, which leads to secretion of pro-inflammatory cytokines, nitric oxide, and eicosanoids (50). In splenic follicular B cells from ChAT^BAC^-eGFP transgenic mice, LPS induces ChAT-GFP expression and increases ACh production (41). These results along with the aforementioned effects of SAC suggest that stimulation of TLRs on B cells activates cholinergic activity by enhancing ACh synthesis. Furthermore, ACh produced by ChAT^+^ B cells has been shown to reduce peritoneal neutrophil recruitment during sterile endotoxemia, suggesting the role for B cell-derived ACh in the regulation of innate immunity (41).

##### DCs and Macrophages

As described, LPS upregulates ChAT mRNA expression in bone marrow-derived DCs, but elicits no apparent effects on ChAT mRNA expression in peritoneal macrophages from C57BL/6J mice (36). Reardon et al. (41) found that induction of ChAT expression in murine macrophages and DCs by LPS elicits MyD88-dependent signal transduction in a cell-intrinsic manner.

#### Activation of the Cholinergic System in Immune Cells

##### Lipopolysaccharide

**Figure 2:**
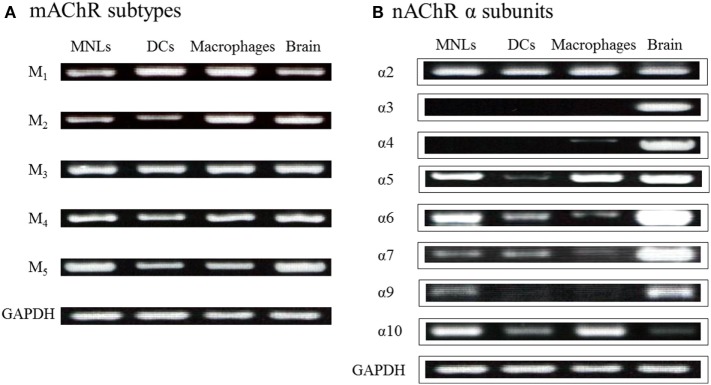
Expression of genes for mAChRs and nAChR α subunits in immune cells from C57BL/6J mice. **(A)** mRNA expression of mAChR subtypes detected using reverse transcription-polymerase chain reaction. MNLs, mononuclear leukocytes; DCs, dendritic cells. GAPDH, glyceraldehyde-3-phosphate dehydrogenase. **(B)** mRNA expression of nAChR α subunits. Arranged from study by Kawashima et al. (36).

As described, among innate immune cells, DCs and macrophages have the potential for ChAT-catalyzed ACh synthesis upon activation with LPS (36–38, 41). LPS and TLR agonists induce expression of ChAT mRNA and enzyme protein in DCs (36, 41) and macrophages (41). MyD88-dependent TLRs are involved in LPS-induced ChAT expression in DCs and macrophages (41). Because DCs and macrophages express all five M_1_–M_5_ mAChR subtypes (36) and various nAChR subunits (Figure 2) (36), these findings suggest the possibility that activation of MyD88-dependent TLRs by LPS upregulates cholinergic activity in macrophages and DCs. Furthermore, ACh synthesized in these cells should act in autocrine/paracrine fashion on their own nAChRs and mAChRs and play a role in regulation of innate immune responses by modulating cytokine production, such as tumor necrosis factor (TNF)-α and interleukin (IL)-2.

##### Antigen Presentation

Phytohemagglutinin and ConA upregulate ChAT expression and ACh synthesis in T cells, which indicates that antigen presentation between naïve CD4^+^ T cells and antigen-presenting cells (APCs), including DCs and macrophages, enhances cholinergic activity in immune cells [see reviews in Ref. (11, 12, 16, 33, 47)]. Recognition by the TCR/CD3 complex of an antigen presented on major histocompatibility complex receptors on APCs triggers activation of PLCγ and Ca^2+^ release from endoplasmic reticulum (ER) in T cells (51, 52). Depletion of ER Ca^2+^ stores results in sustained Ca^2+^ influx through Ca^2+^-release activated Ca^2+^ (CRAC) channels, leading to activation of Ca^2+^-sensitive transcriptional factors, including nuclear factor of activated T cells, which promotes expression of cytokine genes critical for immune responses (53, 54). As described, PHA also upregulates AChE and M_5_ mAChR expression along with ChAT (33, 47, 55). This suggests that ACh released from T cells and APCs (DCs and macrophages) act on their own mAChRs and nAChRs affecting autocrine/paracrine pathways, leading to modification of immune function.

The functions of M_5_ mAChRs are not yet well defined at peripheral autonomic nerve effector junctions, the central nervous system, or immune cells. However, the observations that both antigen-specific IgG_1_ and pro-inflammatory cytokine production are decreased in the M_1_/M_5_-KO mice and that M_5_ mAChRs are upregulated by PHA and ConA suggest that M_5_ mAChRs in immune cells are involved in positive regulation of immune function (33, 47, 56). Consistent with that idea, antigen presentation between naïve CD4^+^ T cells and DCs upregulates T cells expression of ChAT mRNA, ChAT activity, ACh, AChE, and M_5_ mAChR, thereby enhancing cholinergic activity [see reviews in Ref. (11, 12, 33, 47)].

### AChE and ChE

Both AChE and BuChE hydrolyze ACh into choline and acetate to terminate its activity at synapses. In the brain, AChE is mostly found within synaptic clefts between neurons (57, 58), while BuChE is mainly located outside the synaptic cleft and in glial cells (59, 60). Both AChE and BuChE are present at the mouse neuromuscular junction, but exhibit different localization patterns. AChE activity is present in both the primary cleft and in the secondary folds, while BuChE activity appears to be concentrated in structures resembling subsynaptic folds (61). AChE is also found within erythrocytes, but its physiological function there is unknown. BuChE is found primarily in plasma, liver, and the neuromuscular junction [see a review in Ref. (12)].

The decay time constants of focally recorded miniature endplate currents caused by ACh at the neuromuscular junction are 1.04 and 5.4 ms in wild-type (WT) and AChE-KO mice, respectively (62). It is important to note the differences in the inactivation rates and processes between ACh and other neurotransmitters, such as norepinephrine (NE) and epinephrine (EPI). Whereas the action of ACh is terminated within a few milliseconds through enzymatic breakdown, the actions of NE and EPI last much longer until reuptake into nerve terminals and surrounding tissues, and the diffusion decrease their concentrations within the synaptic cleft to subthreshold levels. As a consequence, to have a physiological action, non-neuronal ACh must be released into a microenvironment forming synapse-like structures, such as during antigen presentation and cell-to-cell interaction involving cell adhesion molecules.

AChE is expressed ubiquitously in mouse lymphocytes, DCs, and macrophages (36), while human blood MNLs, CEM human leukemic T cells, and Daudi B cells all express various types of AChE mRNA [see a review in Ref. (10)]. In addition, upregulation of AChE activity by PHA is detected in normal peripheral blood human lymphocytes and in leukemic T cell lines (55). These findings indicate that T cell activation *via* TCR/CD3-mediated pathways enhances expression of cholinergic elements, including ChAT and AChE, within T cells. However, the physiological function of AChE in immune cells has yet to be investigated.

Rivastigmine, which inhibits both AChE and ChE activities, relieves the clinical symptoms and spatial memory deficits in mice with autoimmune encephalomyelitis (EAE) (63). Rivastigmine also decreases the reactivity of encephalitogenic T cells and the production of TNF-α, interferon (IFN)-γ, and IL-17 cytokines in the EAE mouse. All of these effects are abolished by α-BTX, an α7 nAChR antagonist, which suggests that the effects are induced by ACh acting on α7 nAChRs after its levels were increased due to AChE and BuChE inhibition (63).

It has been suggested that functional defects in nAChRs on immune cells contribute to the etiology of inflammatory bowel diseases (IBD), as smoking modifies the development and progression of IBD (64, 65) and immunosuppressants are sometimes effective in patients with IBD (66–68). In addition, AChE-targeting microRNA-132 (miR-132) exhibited some potential to attenuate inflammation by reducing AChE levels in immune cells (69).

### AChRs

Early functional and binding studies revealed the presence of both mAChRs and nAChRs on immune cells [see reviews in Ref. (9, 11, 12, 16)].

#### mAChR Subtypes

The diversity of mAChR functions prompted investigation of their molecular basis using cloning techniques and led to the identification of five distinct mAChR subtypes (M_1_–M_5_) (70–72). These five mAChRs have been divided into two groups based on their functional coupling. The M_1_, M_3_, and M_5_ subtypes are coupled to pertussis toxin-insensitive G_q/11_ proteins, which mediate activation of PLC activity. Upon activation of these mAChR subtypes, PLC hydrolyzes phosphatidylinositol 4,5-bisphosphate, leading to the formation of inositol 1,4,5-trisphosphate (IP_3_) and diacylglycerol. These products then act as second messengers by, respectively, mobilizing Ca^2+^ from intracellular stores and activating PKC (73). The M_2_ and M_4_ subtypes are coupled to pertussis toxin-sensitive G_i/o_ protein, which mediates inhibition of adenylate cyclase, and thus a decrease in cAMP formation, upon activation (74, 75).

##### mAChR Expression in Immune Cells

All five mAChRs subtypes have been detected in lymphocytes, macrophages, and DCs from humans, mice, and rats (Figure 2A) [see reviews in Ref. (16, 36, 76)]. As mentioned, PHA and SAC selectively upregulate expression of M_5_ mAChR mRNA in CEM human leukemic T cells and Daudi B cells, respectively (33, 47). Expression of other mAChR subtypes is not affected in either cell line. Stimulation with PMA plus ionomycin upregulates expression of both M_3_ and M_5_ mAChR mRNA in these cell lines (47). These findings show that immunological stimulation leads to M_5_ mAChR gene expression in lymphocytes and suggest a role for M_5_ mAChRs in the regulation of immune function.

Qian et al. (77) showed that activation of murine splenic T cells for differentiation using monoclonal antibodies against CD3 and CD28 modifies the expression patterns and intensities of mAChRs. These findings suggest the possibility that the expression patterns and intensities of mAChR expression in immune cells may vary among individuals, depending on their immunological status.

#### nAChRs

Nicotinic AChRs are located on the plasma membranes of skeletal muscle cells, neurons, and non-neuronal cells. Activation of nAChRs elicits membrane depolarization and excitation due to a rapid increase in membrane permeability to Na^+^, K^+^, and Ca^2+^. The molecular cloning of nAChR subunits (78) prompted a series of proteomic and genomic investigations of the various nAChR subunit proteins. At present, 10 α (α1–10), 4 β (β1–4), γ, δ, and ε nAChR subunits have been detected through molecular cloning. The α subunits can be subdivided according to their sensitivity to α-BTX: α1, α7, and α9 are α-BTX-sensitive, while α2–α6 are α-BTX-insensitive. nAChRs exist as pentamers composed of 1–5 different subunits. At least two copies of the α subunit are always present among these subunits, and multiple ACh binding sites are formed at the interface of each α subunit and a neighboring subunit.

nAChRs are classified into muscle and neuron types based on their expression sites. Muscle type nAChRs are expressed mainly in skeletal muscle and contain four different subunits within the pentameric complex: (α1)_2_/β1/δ*/*γ in embryonic and denervated muscle, and (α1)_2_/β1/δ*/*ε in innervated adult muscle. By contrast, neuron type nAChRs expressed in the nervous system and non-neuronal cells, including immune cells, are composed of only α (α2–α7, α9, and α10) and β (β2–β4) subunits. The α8 subunit is found only in the visual areas of the avian brain (79). nAChRs consisting of α3β2 and α3β4 are abundantly expressed in peripheral ganglia, while α4β2 subunits are expressed in the brain (80). Within the mammalian brain, about 80% of nAChRs are composed of α4β2 subunits, and 10–15% are composed of α7 (81). The α7 nAChR gene *(CHRNA7)* is expressed widely in the central and peripheral nervous system, and in non-neuronal cells, and the homomeric α7 nAChR exhibits uniquely high Ca^2+^ permeability upon activation (82, 83). The α9 and α10 subunits were first identified in the mechanosensory hair cells of the rat auditory system (84, 85). The α9 subunit was initially postulated to form a homomeric nAChR. However, subsequent studies identified expression of heteromeric α9α10 nAChR subtypes within the hair cells. Unlike other nAChRs, which mediate excitatory neurotransmission, α9α10 nAChR activation elicits hair cell hyperpolarization evoked by Ca^2+^ entry through the receptor, leading to activation of a small-conductance SK2 Ca^2+^-dependent potassium channel (85).

##### Specific Features Related to α7 nAChRs

###### *Human-Specific* *dupα7 nAChRs*

The α7 subunit gene *CHRNA7* is composed of 10 exons, encoding 146 amino acids (exons 1–4) comprising an N-terminal extracellular domain and 384 amino acids (exons 5–10) comprising three transmembrane (M1–M3) domains, a large intracellular loop (M3–M4 loop), a fourth transmembrane (M4) domain, and a short C-terminal extracellular region (86). Gault et al. (87) found *CHRNA7* exons 5–10 duplicated as the *CHRFAM7A* gene encoding dupα7 nAChR in the human genome and its expression in the brain. Although the α7 nAChR and dupα7 nAChR subunits share the same 384 amino acids comprising the four transmembrane domains of the ligand-gated ion channel transmembrane region and a short C-terminal extracellular region, the dupα7 subunit has a shorter N-terminal extracellular domain than the primal α7 nAChR subunit. Because the extracellular N-terminal region of the α7 nAChR contains multiple loops of agonist binding site (88), its structure suggests that the dupα7 nAChR subunit may lack the recognition sites for ACh and α-BTX. Experiments performed with *Xenopus* oocytes co-injected with various ratios of α7/dupα7 mRNA revealed a graded reduction in functional receptor generation proportional to the α7/dupα7 ratio, as measured based on nicotine-elicited α7 currents (89). These results were confirmed by measurements of α-BTX binding, which suggests that as the proportion of the dupα7 subunit increases, there is a reduction in the number of functional α7 receptors that reach the surface of the oocyte (89). These results are consistent with the findings of Araud et al. (86), who reported that *CHRFAM7A* functions as a dominant negative regulator of α7 nAChR.

Human-specific *CHRFAM7A* transcripts were first discovered in the brain (87), but were later detected in human peripheral blood leukocytes, including MNLs (90), macrophages (90, 91), and monocytic cell lines (90, 92). The role of the dupα7 subunit in the regulation of immune function remains to be determined.

###### *Heteromeric* *α7β2 nAChRs*

Although the α7 subunit has long been postulated to form a homomeric α7 nAChR, recent studies suggest the possibility that α7 and β2 subunits form heteromeric α7β2 nAChR subtypes in the brain (93–95). Differences in the pharmacological and functional properties between naturally occurring α7-containing nAChRs in the brain and those of recombinant homomeric α7 nAChRs prompted studies investigating whether the nAChR α7 and β2 subunits can co-assemble to form a functional heteromeric nAChR channel in *Xenopus* oocytes and cell lines (93–95). Detection of co-expressed rat α7 and β2 subunits in co-transfected TSA201 embryonal kidney cells and SH-EP1 human epithelial cells, and of α7 and β2 subunit mRNAs in the rat cholinergic neurons, confirmed their co-assembly in mammalian cells (93, 96, 97). Furthermore, the co-expression significantly slowed the rate of channel desensitization, compared to homomeric α7 channels, and altered the pharmacological properties of the channels. It thus appears that rat nAChR α7 and β2 subunits have the ability to co-assemble and form functional heteromeric nAChRs (94, 95, 98).

Expression of α7β2 nAChRs was confirmed in the human brain through purification of α7 subunit-containing proteins using α-BTX beads followed by western blotting using an anti-β2 subunit antibody (99). Thomsen et al. (99) also found that upon agonist stimulation, the currents carried by heteromeric α7β2 nAChRs show markedly slower rising and decay phases than homomeric α7 nAChRs in HEK293 cells. At present, no information is available on the expression of α7β2 nAChRs in immune cells. Because T and B cells, DCs, and macrophages all express α7 nAChRs (36, 98, 100), it is possible that α7β2 nAChRs are also expressed in immune cells and are involved in regulating immune function.

###### *Ionotropic and Metabotropic Natures of* *α7 nAChRs*

As described, activation of α7 nAChRs using ACh or nicotine elicits a transient increase of [Ca^2+^]_i_ in neurons and immune cells, though in certain types of immune cells the receptor channel rapidly desensitizes, and no transient increase of [Ca^2+^]_i_ is observed (101). Nevertheless, activation of α7 nAChRs can set into motion more prolonged signaling events operating downstream and leading to modulation of immune cell function. Prompted by these observations, the dual ionotropic/metabotropic nature of α7 receptors has been extensively explored to explain the role of α7 receptors (102).

(1) *Ionotropic pathway*: the Ca^2+^ influx through α7 nAChRs induced by stimulation with ACh and agonists activates a phosphorylation cascade *via* PKC. This in turn activates the PI3K/Akt pathway, which promotes nuclear factor erythroid 2-related factor 2 (Nrf2) translocation to the nucleus and overexpression of heme oxygenase 1, resulting in potent anti-inflammatory effects (67, 102–104).

(2) *Metabolic pathway*: evidence now suggests that α7 nAChRs may interact with G-proteins independently of G-protein coupled receptors (102, 105–108). By showing the binding of G-proteins to G-protein-binding sites located in the M3–M4 loop of α7 nAChRs, King et al. (108) demonstrated that direct coupling of α7 nAChRs to G-proteins makes it possible to elicit downstream Ca^2+^ signaling responses that can persist beyond the expected time course of channel activation. It has been suggested that α7 nAChRs coupled with G_s_, G_q/11_, and G_i/o_ proteins locating in the hippocampus and prefrontal cortex of C57BL/6J mice contribute to the regulation of neurite growth (101, 106). In addition, Razani-Boroujerdi et al. (101) found that a functional TCR/CD3 complex and leukocyte-specific tyrosine kinase are required for the nicotine-induced rise in the intracellular Ca^2+^ concentration ([Ca^2+^]_i_) evoked in the absence of extracellular Ca^2+^ *via* α7 nAChRs in T cells. This suggests an alternative metabotropic pathway leading to induction of Ca^2+^ signaling in human T cells. Agonist binding to α7 nAChRs also may activate Janus kinase 2/signal transducer and activator of transcription 3 signaling cascades independently of Ca^2+^ influx, leading to suppression of nuclear transcription factor kappaB-regulated transcriptional activity in macrophages (67, 102–104).

##### Specific Features Related to *α*9 and *α*10 nAChRs

Hecker et al. (109) reported that ACh, choline, phosphocholine, phosphocholine-modified LPS from *Haemophilus influenzae*, and phosphocholine-modified protein all inhibit ATP-mediated IL-1β release independently of the extracellular Ca^2+^ influx through the channel in human monocytic U937 cells expressing the α7, α9, and α10 nACR subunits, and in rat monocytes *via* nAChR-mediated pathways. Furthermore, using U937 cells, Richter et al. (110) found that choline and phosphocholine inhibit ATP-mediated P2X7 receptor activation and IL-1β release. While choline elicits ionotropic current responses at homomeric α9 nACRs, phosphocholine does not trigger ionotropic responses at either homomeric α9 or heteromeric α9/α10 nAChRs. These findings provide evidence that phosphocholine and their derivatives are able to function as metabotropic agonists for heteromeric α9/α10 nAChRs.

##### nAChR Expression in Immune Cells

Figure 2B shows the mRNA expression for nAChR α subunits in MNLs, DCs, and macrophages from C57BL/6J mice (36). Expression of the α2, α5, α6, α7, α9, and α10 subunits is frequently detected in immune cells, including T cells (16, 76, 111, 112). However, the specific patterns of nAChR subunit expression in particular immune cells are not yet settled. As mentioned, it appears that the patterns and the intensities of nAChR subunit expression vary depending on the immunological status of the cell in question (77). It is noteworthy, however, that data from immunohistochemistry and western blots must be interpreted cautiously due to the lack of the specificity of the commercially available antibodies (113, 114).

### SLURP-1 and -2: Endogenous Positive Allosteric Ligands for α7 and α3 nAChRs

SLURP-1 and -2 are endogenous peptides that act as positive allosteric ligands on α7 and α3 nAChRs, respectively (115–119). The discovery of mutations in the gene encoding SLURP-1 in Mal de Meleda (MdM) patients with a characteristic transgressive palmoplantar keratoderma (120) is drawing major research attention to the capacity of SLURP-1 to serve as an epithelial growth modulator (112, 118–122). In addition, recent studies suggest the possibility that SLURP-1 and -2 are also involved in regulating immune cell function (36, 122–126).

#### Gene Expression for SLURP-1 and -2

SLURP-1 and -2 mRNAs are detected in nearly every organ in the C57BL/6J mouse (123). Gene expression for SLURP-1 and -2 was also detected in MNLs, DCs, and macrophages, but neither the T cell activator Con A nor the DC and macrophage activator LPS modified the levels of their expression (36, 123). This suggests that SLURP-1 and -2 are constitutively expressed in these cells, and their expression is independent of immunological stimulation.

#### Immunoreactive SLURP-1 Expression in Immune Cells

**Figure 3:**
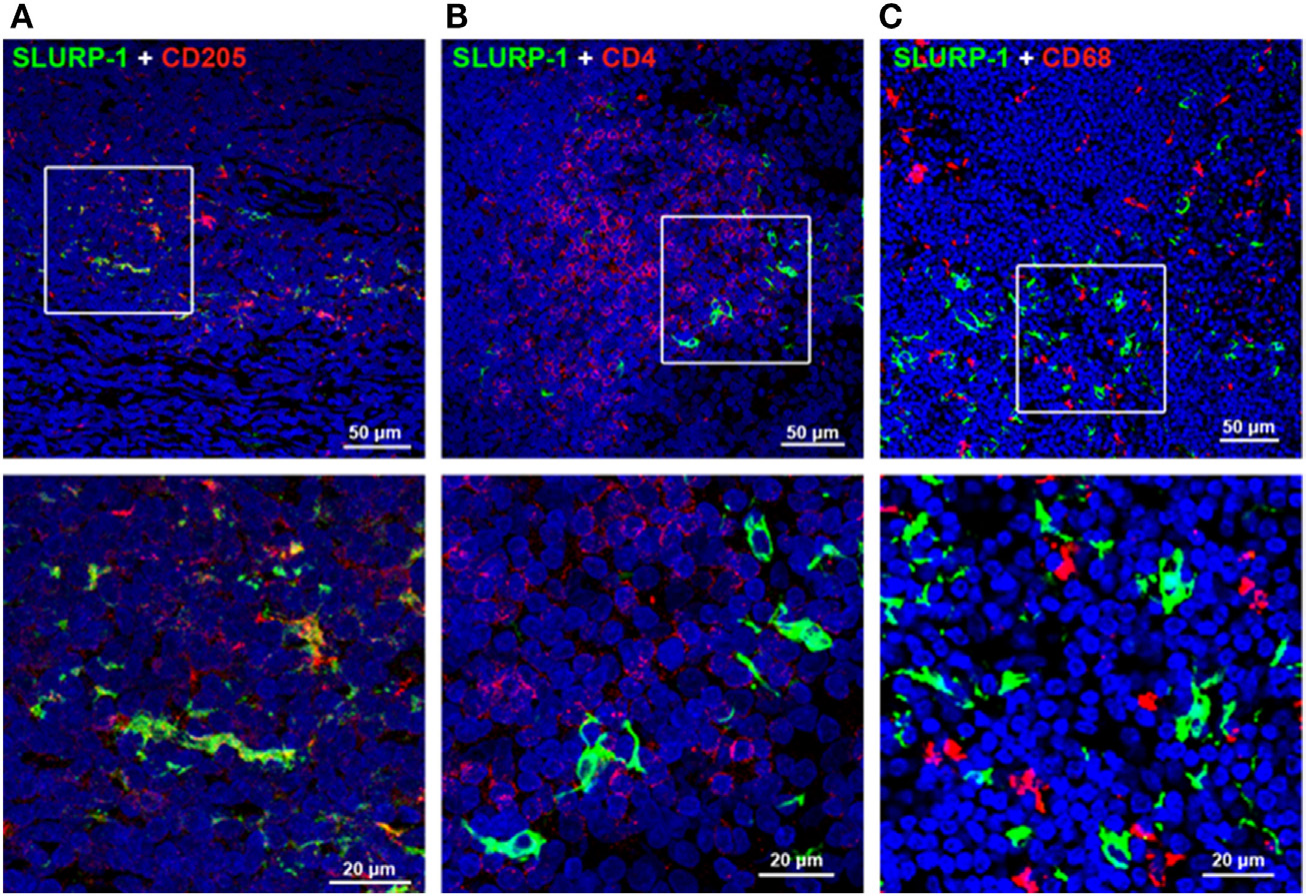
Immunohistochemical staining of SLURP-1, CD205, CD4, and CD68 in human tonsils. **(A)** Merged image showing SLURP-1 (green) and CD205 (red) immunoreactivities in the interfollicular zone. Note that some CD205^+^ cells were also SLURP-1^+^. An enlarged image of the boxed area is shown in the lower panel. **(B)** Merged image showing SLURP-1 (green) and CD4 (red) immunoreactivities in the interfollicular zone. Note that the SLURP-1^+^ cells are surrounded by CD4^+^ T cells. An enlarged image of the boxed area is shown in the lower panel. **(C)** Merged image showing SLURP-1 (green) and CD68 (red) immunoreactivities in the interfollicular zone. Note that the SLURP-1^+^ cells are located in close proximity to CD68^+^ macrophages. An enlarged image of the boxed area is shown in the lower panel. Arranged from data by Fujii et al. (126).

Intense SLURP-1 immunoreactivity (SLURP-1^+^) was detected in DC-like cells residing mainly in the interfollicular zone surrounding the germinal center of human tonsils, and in a few cells scattered within the germinal center (Figure 3) (126). Some SLURP-1^+^ cells in the tonsil interfollicular zone also showed immunopositivity for CD205 (Figure 3A), a marker of mature DCs that mediates efficient antigen presentation (127), and these SLURP-1^+^ CD205^+^ DCs were surrounded by CD4^+^ T cells. These observations support the notion that ACh synthesized and released from T cells and activated macrophages during antigen presentation acts on α7 nAChRs expressed in immune cells, and that SLURP-1 potentiates the ACh activity at α7 nAChRs in both T cells and DCs (16, 126).

## Role of the Cholinergic System in the Regulation of Immune Function

### Roles of mAChRs in the Regulation of Immune Cell Function

Agonist-evoked activation of mAChRs in immune cells evokes a variety of functional and biochemical effects, including enhanced cytotoxicity, increased cGMP and IP_3_ formation, and activation of cell proliferation [see reviews in Ref. (9, 12)]. Stimulation of mAChRs in CEM human leukemic T cells and Daudi human leukemic B cells using ACh, bethanechol, carbachol, or Oxo-M induced a transient rise in the intracellular Ca^2+^ concentration ([Ca^2+^]_i_) followed by extracellular Ca^2+^-dependent [Ca^2+^]_i_ oscillations that persisted for about 10 min (128). In both cell lines, Oxo-M upregulated c-fos mRNA expression in an extracellular Ca^2+^-dependent manner. All these effects induced by mAChR agonists were abolished by atropine, a non-specific mAChR antagonist. This suggests that activation of mAChRs in immune cells triggers nuclear signaling, leading to modification of immune cell function. More recently, Mashimo et al. (54) showed that M_3_ and M_5_ mAChRs play a major role in Oxo-M-induced initial transient increases in [Ca^2+^]_i_ and the following repetitive [Ca^2+^]_i_ oscillations in CEM human leukemic T cells. The [Ca^2+^]_i_ oscillations were blocked by removal of extracellular Ca^2+^ or YM-58483, a CRAC channel blocker, without affecting the Oxo-M-induced initial [Ca^2+^]_i_ transient. Furthermore, CRAC channel blockade abolished Oxo-M-induced c-fos and IL-2 expression. These results suggest that activation of M_3_ or M_5_ mAChRs evokes IP_3_-mediated Ca^2+^ release from intracellular stores, leading to extracellular Ca^2+^ influx through CRAC channels, which generates repetitive [Ca^2+^]_i_ oscillations and, in turn, enhances c-fos and IL-2 gene expression in T cells.

#### M_1_ and M_5_ mAChRs in Cytolytic Activity

Upregulation of M_5_ mAChR mRNA expression in human T and B cells during immunological stimulation strongly suggests that mAChRs, including M_5_, are involved in regulating immune function (33, 47). Thus far, however, there have been few investigations of the roles played by mAChRs in regulation of immune function. The involvement of M_1_ mAChRs in the differentiation of CD8^+^ T cells into cytolytic T cells was first postulated by Zimring et al. (129), but it was later found that neither M_1_ nor M_5_ mAChRs are required for expansion of antigen-specific CD8^+^ T cells, in response to viral infection in C57BL/6 mice (130). On the basis of these observations, the authors concluded that the extent to which one can draw a generalized conclusion that M_1_ and M_5_ mAChRs are not involved in antiviral immunity mediated by CD8^+^ T cells, depends upon issues of antigen strength, genetic background, and receptor redundancy (130).

#### M_1_ and M_5_ mAChRs in Antibody and Cytokine Production

Fujii et al. (56) investigated the roles of M_1_ and M_5_ mAChRs in the regulation of immune function by immunizing combined M_1_ and M_5_ mAChR gene-knockout (M_1_/M_5_-KO) and WT mice with ovalbumin (OVA). One week after the immunization, serum concentrations of anti-OVA-specific IgG_1_ in the M_1_/M_5_-KO mice were significantly lower than in WT mice, though the serum concentrations of anti-OVA-specific IgM did not differ between the two genotypes. Spleen cells from M_1_/M_5_-KO mice activated with OVA secreted significantly lower amounts of TNF-α, IFN-γ, and IL-6 than those from WT mice. These observations suggest that M_1_ and/or M_5_ mAChRs contribute to the regulation of pro-inflammatory cytokine production related to adaptive immunity.

### Roles of nAChRs in the Regulation of Immune Function

Among the various nAChRs, α7 nAChRs are drawing attention because their stimulation attenuates the synthesis and release of the pro-inflammatory cytokine TNF-α in LPS-activated macrophages, leading to modulation of inflammatory and immune responses (15, 131, 132).

#### Upregulation of Antibody and Pro-Inflammatory Cytokine Production in *CHRNA7* Knockout (α7-KO) Mice

Fujii et al. (133) observed a significantly higher serum anti-OVA-specific IgG_1_ levels in α7-KO than WT C57BL/6J mice 2 weeks after immunization with OVA. Moreover, antigen-stimulated spleen cells from α7-KO mice produced significantly greater amounts of TNF-α, IL-6, and IFN-γ responsible for antibody class switch induction to IgG_1_ than those from WT mice. These observations suggest the involvement of α7 nAChRs in regulating pro-inflammatory cytokine production, leading to modification of antibody production. In line with these findings, a recent study showed that nicotine attenuates production of TNF-α, IL-1β, and IL-12 in murine bone marrow-derived monocytes *via* α7 and α9 nAChR-mediated pathways (134). In addition, activation of B cells with anti-CD40 antibody elicited a greater proliferative response in α7-KO mice than in the WT, and the suppression of α7 nAChRs with methyllycaconitine (MLA) evoked a greater proliferative response in B cells stimulated either with anti-CD40 antibody or antibody against B cell receptor (135–138), suggesting a role for α7 nAChRs in downregulation of B cell proliferation. Taken together, the above findings suggest the possibility that a higher serum antigen-specific IgG_1_ concentration observed in α7-KO mice (133) can be ascribed to the upregulation of pro-inflammatory cytokine production and the increased number of B cells in α7-KO mice (135–138).

#### Roles of nAChRs in IL-2 Production and Proliferation of T Cells

MOLT-3 human leukemic T cells and cultured spleen cells from C57BL/6J mice constitutively produce amounts of ACh sufficient to elicit autocrine changes in [Ca^2+^]_i_ and upregulation of IL-2 mRNA and protein expressions (139). Mecamylamine, a nAChR inhibitor, suppressed the [Ca^2+^]_i_ transients, IL-2 release, and cell proliferation. These findings indicate that T cells utilize ACh as a tool to interact with one another and that autocrine ACh-activated nAChRs are involved in regulating immune cell functions such as cytokine synthesis and cell proliferation.

Methyllycaconitine, a specific α7 nAChR antagonist, did not suppress the above described autocrine ACh-induced changes in [Ca^2+^]_i_, suggesting the involvement of nAChRs other than α7 nAChRs in the [Ca^2+^]_i_ transients in freshly isolated spleen cells and resting MOLT-3 cells leading to upregulation of IL-2 production (139). In fact, Qian et al. (77) detected the expression of α7 nAChR mRNA in CD4^+^ and CD8^+^ T cells only after the activation *via* TCR/CD3 cross-linking, but not in freshly isolated CD4^+^ and CD8^+^ T cells. Taken together, these findings suggest the possibility that the pattern of nAChR subtype expression is variable depending on the immunological status. Furthermore, nicotine modified IFN-γ and IL-17 production in T cells activated with TCR/CD3 cross-linking (77, 140). These findings suggest that nAChRs including α7 nAChR contribute to immunomodulation through modification of T cell differentiation by altering cytokine production.

### Roles for SLURP-1 in the Regulation of Immune Cell Function

#### T Cell Activation

T cell activation with anti-CD3/anti-CD28 mAbs of peripheral blood MNLs isolated from MdM patients with SLURP-1 mutation showed a defect in their proliferative response (125). Moreover, addition of WT recombinant SLURP-1 (rSLURP-1) to cultures of T cells from MdM patients restored the normal T cell activation response, showing that SLURP-1 plays a key role during normal activation of T cells induced by immunological stimulation enhancing the actions of ACh *via* α7 nAChRs (125).

#### ACh Synthesis in T Cells

Recombinant SLURP-1 increases ChAT gene expression and the ACh content in MOLT-3 human leukemic T cells and human peripheral blood MNLs, and these effects are abolished by the α7 nAChR antagonist MLA. This suggests that, working as a positive allosteric ligand, SLURP-1 activates cholinergic transmission by potentiating ACh synthesis and its action on α7 nAChRs, thereby facilitating functional development of T cells (126). rSLURP-1 induces a slight but significant attenuation of cell growth in peripheral blood MNLs and MOLT-3 cells and that is abolished by MLA. These findings support the notion that SLURP-1 acts as a key modulator of T cell activity.

#### Effects of SLURP-1 and -2 on Immune Cells

Chernyavsky et al. (122) found that rSLURP-1 decreases production of TNF-α in CEM human leukemic T cells, downregulates IL-1β and IL-6 secretion in U937 macrophages, and moderately upregulates IL-10 production in these immune cells. rSLURP-2 downregulates TNF-α and IFN-γ-receptors in CEM cells and reduces IL-6 production in U937 macrophages. These results demonstrate that SLURP-1 and -2 exert anti-inflammatory effects on T cells and macrophages.

### Roles for α7 nAChRs in T Cell Differentiation

**Figure 4:**
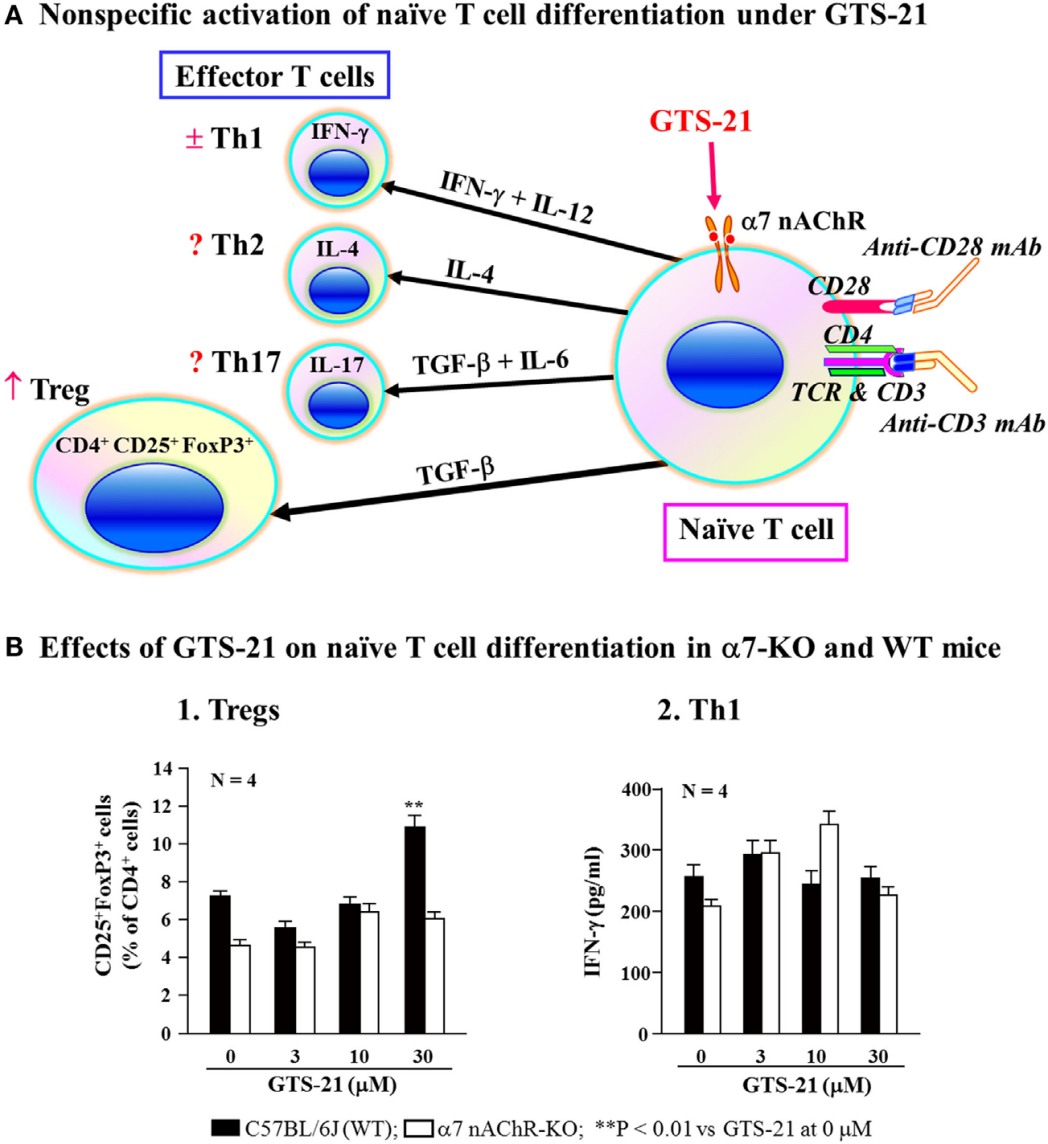
Role for α7 nAChRs during differentiation of naïve T cells. **(A)** Schematic drawing of non-specific activation of naïve T cell differentiation *via* T cell receptor and CD28 in the presence of GST-21, a partial agonist for α7 nAChRs. Differentiation of naïve CD4^+^ T cells into effector T cells (Th1, Th2, and Th17) and regulatory T cells (Tregs) is dictated by the presence of cytokines shown on the respective arrows (142, 143). GTS-21 may facilitate the expression of transcription factor FoxP3 or TGF-β leading to Treg expansion. IFN-γ, interferon-γ; IL-4, interleukin-4; IL-6, interleukin-6; IL-12, interleukin-12; IL-17, interleukin-17; TGF-β, transforming growth factor-β. **(B)** Effects of GST-21 on naïve T cell differentiation in α7 nAChR-deficient (α7-KO) and wild-type (WT) mice. Note that GTS-21 upregulated differentiation into Tregs in the WT, but not α7-KO mice. Th1 differentiation was not affected by GTS-21 in the both genotypes. Arranged from study by Kawashima et al. (141).

As described, α7 nAChRs on immune cells are involved in regulating the synthesis of various cytokines (16, 131, 133). On the basis of those findings, Kawashima et al. (141) used α7-KO and WT mice to investigate the involvement of α7 nAChRs on naïve CD4^+^ T cells in the regulation of their differentiation to CD4^+^ CD25^+^ FoxP3^+^ regulatory T cells (Tregs). Non-specific activation of naïve CD4^+^ T cell differentiation in culture using anti-TCR and anti-CD28 mAbs in the presence GTS-21, a partial α7 nAChR agonist, upregulated the generation of Tregs from WT spleen cells, but not from α7-KO cells (Figure 4). On the other hand, assays of IFN-γ in the culture media suggested that Th1 differentiation was not affected by α7 nAChR activation. This suggests that when naïve CD4^+^ T cells are directly stimulated *via* their TCR/CD3 complex, activation of α7 nAChRs on the T cells leads to upregulation of Treg and that α7 nAChRs are also involved in regulating adaptive immunity.

Studies examining the effects of adoptive transfer of antigen-specific Tregs have demonstrated their contribution to the protection and recovery of an animal model of autoimmune EAE (144). However, the availability of adequate numbers of antigen-specific Tregs for adoptive transfer is difficult to achieve and is one of the major limitations of its clinical application. For treatment of autoimmune diseases, it would be useful to develop procedures in which immune cell cholinergic activity was used to facilitate Treg differentiation from naïve CD4^+^ T cells. Conversely, suppression of Treg differentiation through manipulation of cholinergic activity in immune cells could potentially be of great help in cancers therapy.

## Inflammatory Reflex in the Context of the Immune Cell Cholinergic System

### Inflammatory Reflex

The inflammatory reflex involves the nervous systems and humoral factors that operate to protect the body from infection and inflammation. Pro-inflammatory cytokines generated by immune cells in response to invading pathogens trigger a series of reflex responses. The inflammatory reflex loop consists of two major components: (1) a sensory afferent signaling pathway and (2) a motor efferent signaling pathway. The afferent signaling pathway conveys peripheral signals to the brain *via* the afferent sensory vagus nerve and humoral routes. After the signals are processed in the brainstem, the central nervous system transmits signals to the periphery *via* (1) humoral pathways that involve activation of the hypothalamic–pituitary–adrenal axis, leading to the release of glucocorticoids with anti-inflammatory actions (13), (2) neural pathways involving activation of the sympathetic nervous system (13, 14), and (3) the efferent vagus nerve (16, 145–147). Here, we will mainly touch on the efferent signaling pathways of the inflammatory reflex in the context of the immune cell cholinergic system.

### Background of an Anti-Inflammatory Reflex

Intraperitoneal injection of IL-1β, a pro-inflammatory cytokine, induces fever that is dependent on intact afferent vagus neurons (148). In a rat model of LPS-induced septic shock, electrical stimulation of the efferent vagus nerve protected animals from endotoxemia and attenuated the increase in serum and liver TNF-α levels (149). In addition, ACh and nicotine abolished the LPS-induced release of TNF-α from cultured human macrophages. Electrical stimulation of the vagus nerve also inhibited LPS-induced TNF-α synthesis in WT mice, but failed to inhibit TNF-α synthesis in α7-KO mice, suggesting that α7 nAChRs are essential for inhibiting cytokine synthesis *via* a cholinergic anti-inflammatory pathway (131, 150–152).

Following the observations that splenectomy and selective abdominal vagotomy abolished the anti-inflammatory effects of both vagus nerve stimulation and nicotine, the spleen was identified as an essential target of the cholinergic anti-inflammatory pathway involved not only in inflammatory signal generation in the afferent vagus nerve but also vagally mediated inhibition of cytokine production in macrophages (153–155).

Although the attenuation of LPS-induced septic shock by efferent vagal stimulation suggests the involvement of the vagus nerve in the anti-inflammatory reflex (150), the pathways and mechanisms that carry efferent inhibitory signals suppressing the synthesis and release of pro-inflammatory cytokines in the spleen are not yet settled. There is no neuroanatomical evidence for a direct parasympathetic or vagal nerve supply to any immune organ (156). The currently available evidence indicates that all primary and secondary immune organs receive innervation only by sympathetic postganglionic neurons [see a review by Nance and Sanders (14)]. Prevertebral sympathetic ganglia associated with the celiac-mesenteric plexus provide major sympathetic input to the spleen, and the splenic nerve is the final common pathway for neural input to the spleen (14, 157).

### Currently Proposed Efferent Signaling Pathways in the Anti-Inflammatory Vagal Reflex

#### Anti-Inflammatory Vagal Reflex Mediated by Noradrenergic Splenic Nerve and ChAT^+^ T Cells in the Spleen

Because the efferent vagus nerve that should carry the efferent signals terminates in the celiac ganglion, Rosas-Ballina et al. (15) postulated that the efferent signals carried by the efferent vagus nerve are transmitted to the postganglionic sympathetic splenic nerve innervating the spleen in the celiac ganglion. This would activate the splenic nerve to release NE within the spleen, which would promote Ach release from a subset of CD4^+^ T cells that relay the neural signal to other immune cells—e.g., through activation of α7 nAChRs on macrophages, resulting in inhibition of the synthesis and release of pro-inflammatory cytokines, including TNF-α. Consistent with those ideas, they found that electrical vagus stimulation elevated ACh concentrations in the spleen, and attenuated serum TNF-α levels during endotoxemia. Their observations further confirmed that T cells mediate the inflammatory reflex. They found that vagus nerve stimulation did not diminish serum TNF-α concentrations during endotoxemia in nude mice lacking functional T cells, but that the transfusion of a subset of ChAT^+^ T cells from normal mice to the nude mice restored the ability of vagus nerve stimulation to suppress serum TNF-α concentrations. They, therefore, proposed that efferent vagus nerve signals are transferred to ChAT^+^ T cells *via* NE released from the splenic nerve in the spleen, leading to elevation of ACh synthesis through activation of β-adrenoceptors on the ChAT^+^ T cells. The elevated ACh in turn acted on α7 nAChRs to suppress synthesis and release of TNF-α in macrophages (15).

#### Anti-Inflammatory Vagal Reflex Mediated by Recruited ChAT^+^ T Cells and NE Released from Sympathetic Nerve Terminals in the Spleen

Based on the observation that electrical stimulation of the vagus nerve did not elicit action potentials in the splenic nerve (158), Martelli et al. (159) proposed that efferent vagal stimulation and/or antidromic vagal afferent activation elicits lymphocyte mobilization to the spleen from a deposit of lymphocytes (vagal target). Then within the spleen, ACh-synthesizing CD4^+^ T cells among the mobilized lymphocytes release ACh to activate α7 nAChRs on the sympathetic nerve terminals leading to NE release. NE released from sympathetic nerve terminals acts on β_2_-adrenoceptors on macrophages to suppress TNF-α synthesis and release [see reviews in Ref. (13, 14)]. However, considering the extreme enzymatic fragility of ACh, it is essential to confirm the formation of synapse-like structures between ACh-synthesizing CD4^+^ T cells and sympathetic nerve terminals in the spleen, and between vagus nerve terminals and the vagus target. It is also necessary to demonstrate α7 nAChR expression in the sympathetic nerve terminals in the spleen and their functional release of NE.

#### A Possible Involvement of the Cholinergic System in Immune Cells and SLURP-1 in Inflammatory Reflex

SLURP-1 acts as a positive allosteric ligand for α7 nAChRs (117, 118) and increases the potency and efficacy of ACh at α7 nAChRs more than 2.5 times (117). SLURP-1 co-localizes with subsets of sensory neurons containing substance P (SP) and calcitonin gene-related peptide (CGRP) (160), which have been detected among afferent vagus nerve fibers (161, 162). In the white pulp of the spleen, SP^+^ nerve fibers have been identified within the marginal zone and the outer regions of the periarteriolar lymphoid sheaths, which are filled with T cells (163). In mice, CGRP^+^ nerve fibers have been detected in the spleen and lymph nodes (164, 165). Furthermore, the significant increase in splenic CGRP levels seen during the initial swelling phase of antigen-induced arthritis in mice suggests inflammation-induced release of CGRP from the sensory neurons (166). These findings provide evidence for splenic innervation by sensory neurons containing both SP and CGRP, and suggest the possibility of co-localization of SLURP-1 in the SP and CGRP nerve fibers innervating the spleen.

**Figure 5:**
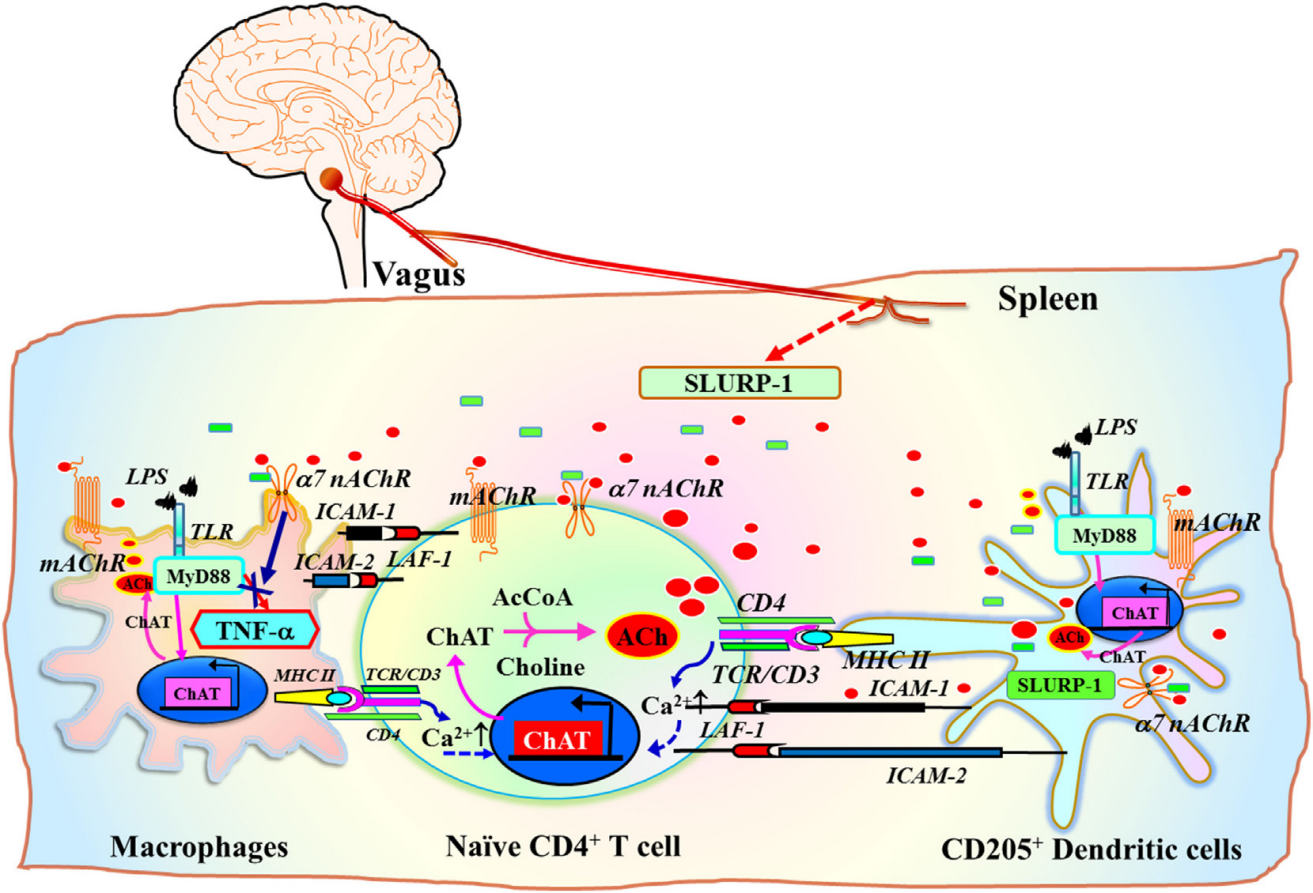
Schematic drawing of the efferent signaling pathway of the inflammatory reflex in the context of the cholinergic system in immune cells. ChAT^+^ T cell interaction with antigen peptides loaded on MHC class II on dendritic cells (DCs) or macrophages *via* the TCR/CD3 complex, CD80/CD86 (B7) co-stimulatory molecules with CD28, and ICAM-1/ICAM-2 with LFA-1, increases the synthesis and release of ACh from T cells [see a review by Fujii et al. (16)]. Inflammatory mediators induce the release of the positive allosteric α7 nAChR ligand SLURP-1 from SP/CGRP-containing sensory fibers. Efferent inflammatory reflex signaling *via* the vagus nerve may also induce released SLURP-1 from SP^+^/CGRP^+^ fibers in the spleen. This released SLURP-1, as well as SLURP-1 released from CD205^+^ mature DCs, potentiates the action of ACh from ChAT^+^ CD4^+^ T cells at α7 nAChRs on macrophages, thereby suppressing synthesis and release of tumor necrosis factor (TNF)-α. The model proposed here is from reviews by Kawashima et al. (111) and Fujii et al. (16). Green rectangles depict SLURP-1. Red ellipses depict acetylcholine. AcCoA, acetyl coenzyme A; ICAM-1, intercellular adhesion molecule-1; ICAM-2, intercellular adhesion molecule-2; LFA-1, lymphocyte function-associated antigen-1; LPS, lipopolysaccharide; MHC II, major histocompatibility complex class II; SLURP-1, secreted lymphocyte antigen-6/urokinase-type plasminogen activator receptor-related peptide-1; TCR, T cell receptor; TLR, toll-like receptor.

A subset of CD205^+^ DCs located within the marginal zone of human tonsils contain immunoreactive SLURP-1 and are surrounded by CD4^+^ T cells and CD68^+^ macrophages (126) (Figure 3C). Detection of SLURP-1 in human plasma, urine, sweat, saliva, and tears is suggestive of the protein’s stability and mobility (167). Moreover, SLURP-1 potentiates ACh synthesis in T cells (126). It is therefore reasonable to suggest that by potentiating the action of ACh at α7 nAChRs, SLURP-1 released from CD205^+^ DCs during antigen presentation and from SP/CGRP-containing neurons may participate in the inflammatory reflex leading to suppression of TNF-α synthesis in splenic macrophages (Figure 5). Because CD4^+^ T cells can directly interact with splenic macrophages through antigen presentation or cell-to-cell adhesion *via* LFA-1 (CD11a), ACh released from CD4^+^ T cells should act effectively on α7 nAChRs on macrophages in the presence of SLURP-1, leading to the suppression of TNF-α synthesis and release.

## Conclusion

Currently available evidence suggests that the immune cell cholinergic system makes a critical contribution to the regulation of immune function. For example, the cholinergic signals generated by immune cells appear to be triggers of both the initiation and termination of cytokine synthesis (e.g., IL-2 in T cells and TNF-α in macrophages). Furthermore, the evidence raises the possibility that immune function can be modulated by manipulating the cholinergic activity of immune cells. Thus, a fuller understanding of the immune cell cholinergic system could be useful for the development of drugs and therapeutic strategies for the treatment of inflammation-related diseases and cancers.

## Author Contributions

TF, HM, SO, and KK conceived of the review. TF and KK drafted the initial version of the manuscript and refined the contents. MM, YM, SO, and KH contributed to reference analyses, prepared tables and figures, and revised the manuscript, which was progressively edited by TF, MM, SO, and KK. TF, MM, YM, HM, SO, KH, and KK reviewed and approved the final version of this review manuscript.

## Conflict of Interest Statement

The authors declare that the research was conducted in the absence of any commercial or financial relationships that could be construed as a potential conflict of interest.
